# Unique genome-wide transcriptome profiles of chicken macrophages exposed to *Salmonella*-derived endotoxin

**DOI:** 10.1186/1471-2164-11-545

**Published:** 2010-10-08

**Authors:** Ceren Ciraci, Christopher K Tuggle, Michael J Wannemuehler, Dan Nettleton, Susan J Lamont

**Affiliations:** 1 Departments of Animal Science, Iowa State University, Ames, Iowa 50011 USA; 2 Veterinary Microbiology and Preventive Medicine, Iowa State University, Ames, Iowa 50011 USA; 3 Statistics, Iowa State University, Ames, Iowa 50011 USA

## Abstract

**Background:**

Macrophages play essential roles in both innate and adaptive immune responses. Bacteria require endotoxin, a complex lipopolysaccharide, for outer membrane permeability and the host interprets endotoxin as a signal to initiate an innate immune response. The focus of this study is kinetic and global transcriptional analysis of the chicken macrophage response to *in vitro *stimulation with endotoxin from *Salmonella typhimurium*-798.

**Results:**

The 38535-probeset Affymetrix GeneChip Chicken Genome array was used to profile transcriptional response to endotoxin 1, 2, 4, and 8 hours post stimulation (hps). Using a maximum FDR (False Discovery Rate) of 0.05 to declare genes as differentially expressed (DE), we found 13, 33, 1761 and 61 DE genes between endotoxin-stimulated versus non-stimulated cells at 1, 2, 4 and 8 hps, respectively. QPCR demonstrated that endotoxin exposure significantly affected the mRNA expression of *IL1B*, *IL6*, *IL8*, and *TLR15*, but not *IL10 *and *IFNG *in HD 11 cells. Ingenuity Pathway Analysis showed that 10% of the total DE genes were involved in inflammatory response. Three, 9.7, 96.8, and 11.8% of the total DE inflammatory response genes were significantly differentially expressed with endotoxin stimulation at 1, 2, 4 and 8 hps, respectively. The *NFKBIA, IL1B, IL8 and CCL4 *genes were consistently induced at all times after endotoxin treatment. *NLRC5 *(CARD domain containing, NOD-like receptor family, RCJMB04_18i2), an intracellular receptor, was induced in HD11 cells treated with endotoxin.

**Conclusions:**

As above using an *in vitro *model of chicken response to endotoxin, our data revealed the kinetics of gene networks involved in host response to endotoxin and extend the known complexity of networks in chicken immune response to Gram-negative bacteria such as *Salmonella*. The induction of *NFKBIA, IL1B, IL8, CCL4 *genes is a consistent signature of host response to endotoxin over time. We make the first report of induction of a NOD-like receptor family member in response to *Salmonella *endotoxin in chicken macrophages.

## Background

Determining the effects of endotoxin from *Salmonella typhimurium *in chicken macrophages is an in vitro model to characterize the transcription profiles of one important cell type in the chickens' immune response. Endotoxin is a complex lipopolysaccharide (LPS) found in the outer cell membrane of Gram-negative bacteria that is responsible for membrane organization and stability [1] and differs from LPS in that it is a butanol/water extract rather than a phenol/water extract [2]. Endotoxin used in the present study is between 10 and 20% protein and reproducible, hence its complexity better mimics the cell membrane *in vivo*. Recognition of the lipid A and/or the polysaccharide moiety of endotoxin by membrane receptors of monocytes induce a wide variety of cellular responses, including the synthesis of cytokines such as *IL1B, TNF, IL6, IL8 *[3]. Vertebrates have evolved an effective innate immune response to LPS-containing bacteria over evolutionary time. Chickens are much more resistant than mammals to LPS-induced septic shock [4] and respond to LPS with the induction of *IL1B, IL6*, and *IL18 *mRNA [5]. However, few studies have specifically examined the response to the more complex and more relevant immune stimulant, endotoxin, as a model for *in vivo *responses.

Membrane-bound receptors (some Toll-like Receptors; TLRs) and also intracellular receptors such as NOD-like Receptors (NLRs) play key roles in the recognition of pathogen associated molecular patterns (PAMPs) to induce a host response. Both receptor families contain a series of Leucine Rich Repeat (LRR) modules in their ligand recognition domains [6]. Although NLRs have been extensively studied in mammals [7], their regulation in chicken is still to be described

Macrophages play primary roles in both innate and adaptive immunity. In addition to their roles in innate disease resistance, macrophages are versatile cells that can alter the animal's immunological state by producing regulatory molecules such as cytokines, enzymes, and receptors that regulate the adaptive immune response [8]. Cell lines allow better experimental control and reproducibility than primary cultures of macrophages because of the functional uniformity of cell populations [9]. Despite the limited number of studies with chicken macrophages, it is known that they are capable of mediating lymphoid functions [10]. HD11 is an avian myelocytomatosis virus (MC29) transformed chicken macrophage-like cell line [11] that has been extensively studied. For example, LPS induced a significant level of nitric oxide production (NO) in HD11 cells [12]. HD11 cells have been shown to be activated, as measured by NO production, by various doses of LPS by He et al. (2006) [13]. This dose-dependent induction of NO in HD11 cells at 24 hours post stimulation demonstrates involvement in host response mechanisms to microbial infections and responsiveness of HD11 cells to bacterial components.

Gene expression profiling using microarrays is a widely used method to explore biological functions of both host and microorganisms in innate immunity [14,15]. Classifying interconnected and overlapping components of the immune system into subsets, according to their functionality, such as cellular versus humoral immunity or innate versus adaptive immunity, permit the complex immune system to be dissected into distinct areas. Chicken macrophage immune response to strains of avian pathogenic *Escherichia coli *(APEC) and *Mycoplasma synoviae *was previously studied in HD11 cells using the avian macrophage microarray (AMM) with 4906 elements and using the avian innate immunity microarray (AIIM) with 4959 elements [16]. The AMM with 4906 elements has also been used by Bliss et al. (2005) to determine the avian macrophage response to commercial *Salmonella typhimurium *lipopolysaccharide [17]. However, the AMM profiling tool lacked some important elements; for example, replicates of probes for known Toll-like receptor genes were missing. Transcriptional profiling of chicken HD11 cells stimulated with *Salmonella enteritidis *was performed using the AMM array, and the authors reported that most of the DE genes responded at 5 hours post stimulation, with more genes down-regulated than up-regulated [18].

In the present study, a global transcriptome analysis of the HD11 innate immune response was conducted. The HD11 cells were exposed to various doses of ST-798 endotoxin for 1, 2, 4, and 8 hours and the mRNA levels *of IL6, IL8, IL10, IL1B, IFNG*, and *TLR15 *genes were measured by Quantitative RT-PCR and with the *Affymetrix *GeneChip containing 38535 probes. First, we determined the optimum among four endotoxin doses to elicit an immune response in HD11 cells and then performed a microarray experiment. Our results showed a chicken host response to *Salmonella *endotoxin that initiated quickly and significantly, increased in breadth up to 4 hps, and then rapidly approached homeostasis at 8 hps. The data suggest the importance of these early-induced genes in initiating the extensive gene cascade occurring at 4 hours exposure. We classified all significantly differentially expressed genes by their function and compared gene networks at 1, 2, 4, and 8 hours post-stimulation. The large number of genes differentially expressed at 4 hours enabled the elucidation of highly refined gene networks. This study provides a more comprehensive assessment of chicken macrophage response to endotoxin from *Salmonella typhimurium *(one of the most common food-borne pathogen) than the literature published to date, along with other novel findings on specific genes

## Results

### Endotoxin dose of 1 μg/ml consistently induces an immune response in chicken macrophages

**Figure 1:**
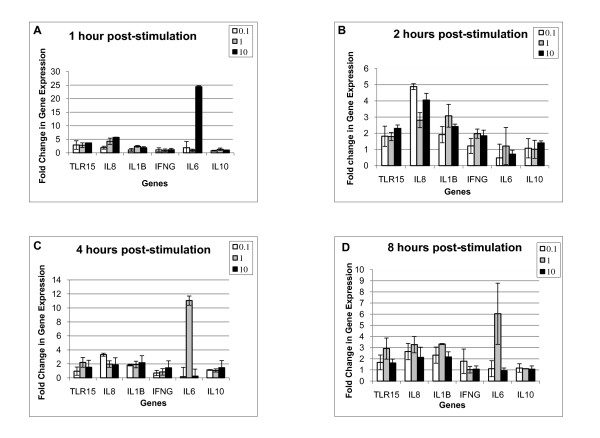
**Endotoxin stimulation of *TLR15, IL8, IL1B, IFNG, IL6 *and *IL10 *gene expression in HD11 cells**. HD11 macrophages were stimulated for 1, 2, 4 and 8 hours with 0.0, 0.1, 1.0, 10.0 μg/ml endotoxin. Data are shown as the fold change in mRNA levels after treatment compared with nontreated cells by QPCR. A: fold change at 1 h post-stimulation (hps); B: fold change at 2 hps; C: fold change at 4 hps; D: fold change at 8 hps. RNA samples were isolated on 3 different days and the QPCR was carried out in triplate.

HD11 cells were stimulated with 0.0, 0.1, 1.0, or 10.0 μg/ml endotoxin for 1, 2, 4, or 8 hours and the differential expression of *IL6, IL8, IL10, IL1B, IFNG*, and *TLR15 *genes was measured by QPCR. Multiple comparison analysis of least squares means (LSmeans) demonstrated that 1 μg/ml of endotoxin was the minimum concentration required to elicit an immune response in HD11 cells, assayed by transcriptional differences in these selected genes (Table 1). Macrophages stimulated with endotoxin expressed significantly higher levels of *IL6, IL8, IL1B*, and *TLR15 *than the non-stimulated (vehicle-treated) macrophages. Cells stimulated with 1.0 μg/ml endotoxin also expressed higher mRNA than cells stimulated with 0.1 μg/ml endotoxin for *IL1B *(*P *< 0.0001) and *IL6 *(*P *= 0.03). Stimulation of cells with endotoxin of all doses induced higher *IL8 *expression than in non-stimulated cells (*P *< 0.0001). Cells stimulated with 1.0 μg/ml endotoxin expressed higher levels of *TLR15 *than non-stimulated cells (*P *= 0.002). *IL10 *gene expression did not change by endotoxin dose. The stimulation time had significant effect on the mRNA levels of all genes assayed by QPCR. The endotoxin dose significantly affected the expression of TLR15, IL1B, IL8 and IL6 (Table 2). Thus, endotoxin stimulation of HD11 macrophages had different impacts on each gene (Table 3). *IL1B, IL8, IL6 *and *TLR15 *gene expression differed by the endotoxin dose, while no *IFNG *or *IL10 *induction was measured after endotoxin stimulation (Fig. 1A). Endotoxin treatment, comparing treated to non-treated cells, had significant or near significant effects on IL1beta and IL8 genes at 2, 4 and 8 hps (Fig. 1B, 1C, 1D).

**Table 1:** Effect of endotoxin dose and time on cytokine expression in HD11 macrophages (P values)

Genes	Time	Dose	Interaction
TLR15	0.026	0.002	0.693
IL1B	< 0.0001	< 0.0001	0.674
IL8	< 0.0001	< 0.0001	0.539
IFNG	< 0.0001	0.376	0.802
IL10	< 0.0001	0.429	0.783
IL6	0.014	0.034	0.018

**Table 2:** Separation of dose effect on HD11 macrophages over time, significance at P < 0.05

Genes	0.0	0.1	1.0	10.0
TLR15	x	xy	y	y
IL1β	x	y	z	yz
IL8	x	y	y	y
IFN	x	x	x	x
IL10	x	x	x	x
IL6	x	x	y	xy

x, y, z = doses not sharing a letter are significantly different at P < 0.05, by Tukey-Kramer Honestly test

**Table 3:** Effect of endotoxin on cytokine expression at 1, 2, 4 and 8 hours, P values.

Genes	1hps	2hps	4hps	8hps
TLR15	0.027	0.290	0.210	0.136
IL8	0.003	0.027	0.027	0.078
IL1β	0.013	0.004	0.063	0.003
IFNG	0.959	0.412	0.425	0.517
IL6	0.034	0.825	0.002	0.251
IL10	0.336	0.555	0.520	0.946

### Transcriptional response of chicken macrophages to S*almonella e*ndotoxin

We used the array to profile the transcriptional response of chicken HD11 cells to endotoxin over time. We found 13, 33, 1761, and 61 genes significantly DE between endotoxin-stimulated (1.0 μg/ml) and vehicle-treated HD11 cells at 1, 2, 4, and 8 hours; respectively (*q *< 0.05). Our results provide a unique and more comprehensive chicken transcriptome profile than current literature.

Comparative analysis of DE genes by Ingenuity Pathway Analysis showed that 10% of the total DE genes are annotated as inflammatory response [19]. Three, 9.7, 96.8, and 11.8% of these inflammatory response genes were significantly affected at 1, 2, 4, and 8 hours, respectively [additional file 1].

The 13 genes responding to endotoxin stimulus at 1 hour exposure were *TNFAIP3 *(Tumor Necrosis Factor, alpha-induced protein 3)[GeneBank: XR_026935], *TNIP2 *(TNFAIP3 interacting protein 2)[GeneBank: NM_001031166], *NFKBIA *(nuclear factor of kappa light polypeptide gene enhancer in B-cells inhibitor, alpha) [GeneBank: NM_001001472], *MRGPRH *(seven transmembrane domain G-protein coupled receptor) [GeneBank: XM_418053], *BTG2 *(B-cell translocation gene 2) [GeneBank: XM_418053], *IL1B *(Interleukin 1 Beta) [GeneBank:NM_204524], *CCL4 *(chemokine (C-C motif) ligand 4) [GeneBank: NM_001030360], *CD83 *(CD83 antigen, activated B cells) [GeneBank: XM_418929], *IL8 *(Interleukin 8) [GeneBank: NM_205498], *CH25 H *(cholesterol 25-hydroxylase) [GeneBank: XM_421660], *TRAF3 *(TNF receptor-associated factor 3 [GeneBank:XM_421378], and *JUN *(jun oncogene) [GeneBank: NM_001031289] genes. Most, if not all, of these 13 genes have key roles in the immune response and were significantly up-regulated (Table 4). The number of significantly DE genes increased from 13 to 33 at 2 hours post stimulation. Four hours after stimulation, 1761 genes were differentially expressed with about 2/3 up-regulated and 1/3 down-regulated [additional file 2]. Interestingly, all stimulated genes at both 1 hps and 2 hps except *LIPG *were still up-regulated at 4 hps, and all but three (*TRAF3, JUN *and *TNIP2*) of the genes stimulated at 1 hps, and all but 4 of the 2 hps up-regulated genes (*DUSP10 *and three un-annotated transcripts), remained elevated at 8 hps, indicating much of the earliest immune response stimulation was still occurring. Clearly, however, the majority of the massive response observed at 4 hps was very transitory, significantly shutting down by 8 hps.

**Table 4:** Fold changes [log2(treated/control)] at 1 hour post-stimulation, *q *values from microarray analysis

Gene name	Accession number	fold change log2(t/c) at 1 hps	*q*-values
TNFAIP3	XR_026935	2.64	4.29E-07
NFKBIA	NM_001001472	2.29	4.93E-05
MRGPRH	XM_423677	2.29	0.0003
BTG2	XM_418053	2.14	0.0005
IL1β	NM_204524	5.65	0.0009
CCL4	NM_001030360	5.65	0.0009
CD83	XM_418929	3.48	0.009
IL8	NM_205498	2.96	0.01
CH25H	XM_421660	2.46	0.02
TRAF3	XM_421378	1.86	0.03
JUN	NM_001031289	1.62	0.03
IL8	NM_205018	3.03	0.03
TNIP2	NM_001031166	1.74	0.03

### Persistent inflammatory response across all time points but a specific anti-microbial response only at 4 hours after endotoxin stimulation

**Figure 2:**
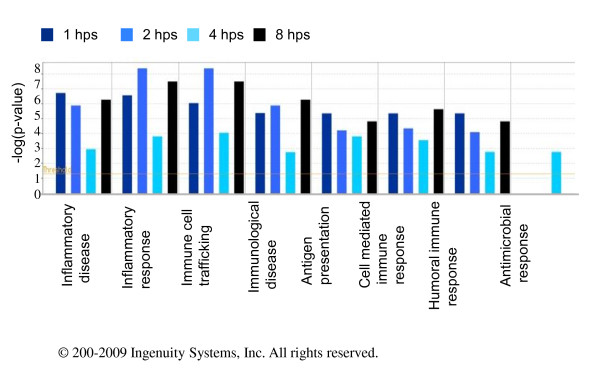
**Significance levels of different immunological functions of chicken HD11 cells stimulated with endotoxin**. Immunological functions at 1, 2, 4, and 8 hours post stimulation*P*-values were calculated by Fisher's exact test using IPA. Threshold was set at P = 0.05 and indicated as -log (p-value) on the Y-axis. X-axis shows immunological function. Experiments carried out in triplicates.

Transcriptional regulation of chicken macrophages changed as a result of endotoxin treatment observed as early as 1 hour post exposure. To explore these changes, we categorized DE genes by function, with an emphasis on immunological functions, and compared the *P-*values for all time points within each functional group using Ingenuity Pathway Analysis (IPA) software (Fig. 2). The significance levels varied across the functional groups. Genes annotated with various types of immune and inflammatory response functions were significantly overrepresented in all gene lists. However, the genes in the "Antimicrobial Response" functional category were differentially expressed only at 4 hps, demonstrating the specific character of the immune response of chicken macrophages to ST-798 endotoxin at 4 hps.

### Genes involved in "immune cell trafficking" networks after endotoxin stimulation

**Figure 3:**
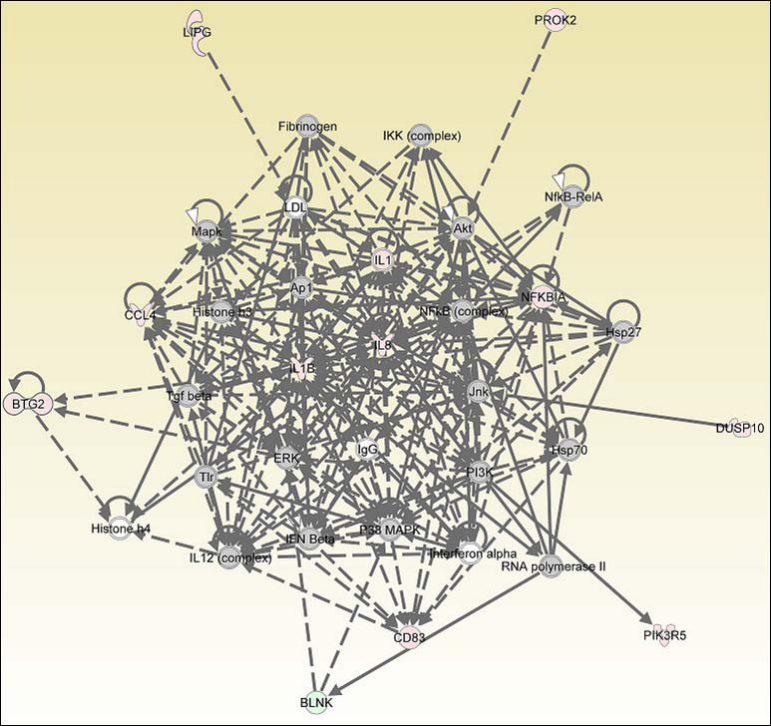
**Gene network analysis of microarray of chicken HD11 cells at 2 hours after endotoxin stimulation**. "Cell-to-cell signaling and interaction, hematological system development and function, immune cell trafficking" gene networks at 2 hps. Red color shows up-regulation and green color shows down-regulation (IPA). Grey molecules are not differentially expressed, but are included to illustrate association with significantly up-regulated genes. Experiments were carried out in triplicate.

**Figure 4:**
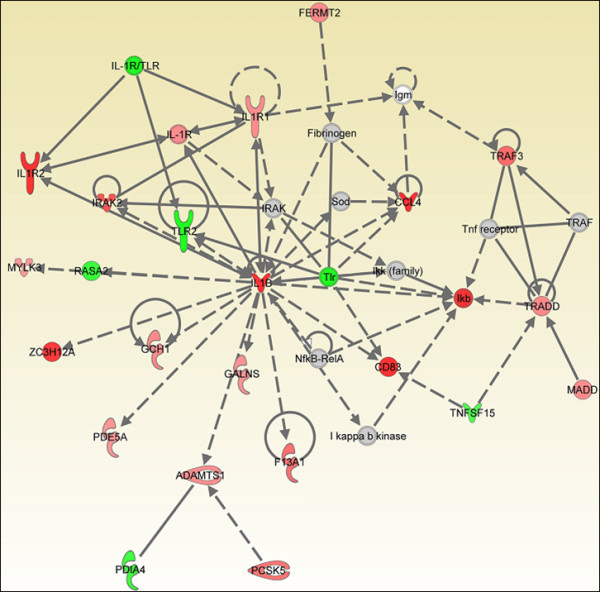
**Gene network analysis of microarray of chicken HD11 cells at 4 hours after endotoxin stimulation**. Experiments were carried out in triplicate.

**Figure 5:**
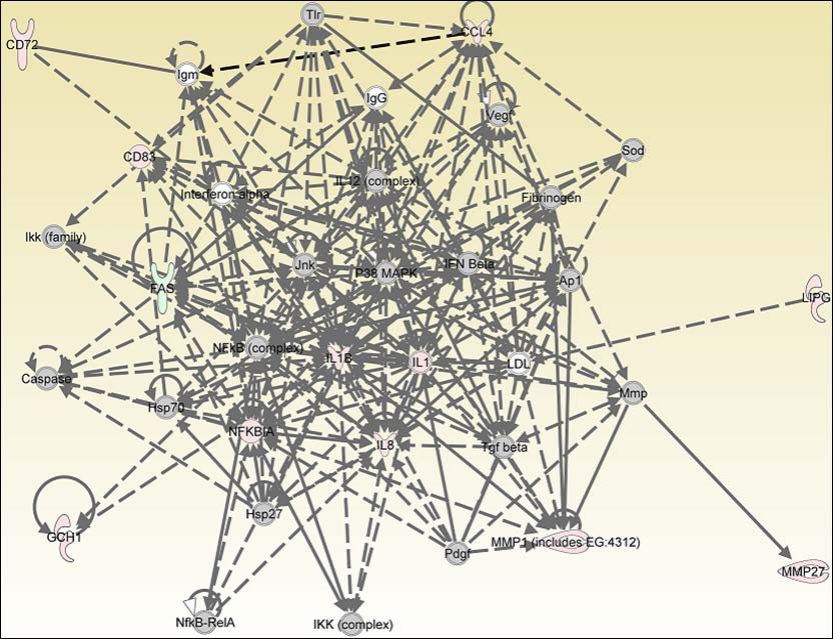
**Gene network analysis of microarray of chicken HD11 cells at 8 hours after endotoxin stimulation**. Experiments were carried out in triplicate.

We then used IPA for comparative gene network analysis (Fig. 3, 4, 5). Ingenuity Pathway Analysis considers all possible interactions between the genes, including the ones that are not in the entered gene list. During the first hour of endotoxin exposure, only 13 genes were significantly up-regulated, which resulted in a network only lightly populated with our DE genes and thus provided little insight (Fig. 3, 5).

The one hour post-stimulation response was the limiting factor in network comparisons because of the small number of differentially expressed genes. Gene networks of "immune cell trafficking" were identifiable at all four time points, however, and therefore were used for comparison of network structure over time. At 1 hps, the *BTG2, IL8, TNIP2 *and *CCL4 *genes were included in the "Cell-To-Cell Signaling and Interaction, Hematological System Development and Function, Immune Cell Trafficking" group according to their function [additional file 3]. *NFKBIA, IL1B, IL8*, and *CCL4 *genes were persistently up-regulated at each time point. *AP1 (JUN) *transcription factor was induced when macrophages were exposed to endotoxin for 1 hour, but this expression profile was not observed at 8 hours exposure. However, an *NFKB *dependent host response was shown by the significant differential expression of *NFKBIA*. Phosphorylation and the subsequent ubiquitination of IKB, the gene product of the *NFKBIA *gene, are known as key processes required for regulating the innate immune system [20]. We observed a significant increase, after endotoxin stimulation at 4 hours, in the mRNA levels of IL-1 receptor-associated kinase 2 (*IRAK2*) which regulates phosphorylation and the genes that are involved in ubiquitination: ubiquitin-conjugating enzyme E2Q family member 2 (*UBE2Q2*), ubiquitin protein ligase E3C (*UBE3C*), ubiquitin-conjugating enzyme E2A (RAD6 homolog) (*UBE2A*), ubiquitin-conjugating enzyme E2, J1 (UBC6 homolog, yeast) (*UBE2J1*), ubiquitination factor E4B (UFD2 homolog, yeast) (*UBE4B*) (Table 5, additional file 2). Because the number of differentially expressed genes increased with time up to 4 hps, we were able to define more precise interactions at that time among the analyzed genes using the Ingenuity Pathway Analysis software (Fig. 4) IL1 receptor family members, *IL1RL2 *(interleukin 1 receptor-like 2) and *IL1R2 *(interleukin 1 receptor, beta) were responsive to 4 hours of endotoxin stimulation relative to untreated cells (fold change = 1.4, 2.2; *q *= 0.039811, 0.001; respectively). We conclude that *IL1B *is a central node in the cellular response network due to its coordination and interactions with other molecules in the network (Fig. 4). The functions of all genes demonstrated in the networks at all time points are indicated in additional file 2.

**Table 5:** Effects of endotoxin on genes involved in ubiquitination at 4 hps.

Gene name	Accession number	Fold change log2(t/c) at 4 hps	*q*-values
IRAK2	NM_001030605	0.82	0.005
UBE2Q2	XM_413740	0.34	0.045
UBE3C	NM_001030967	0.35	0.049
UBE2A	NM_204865	0.37	0.036
UBE2J1	NM_204763	0.44	0.028
UBE4B	XM_417607	0.73	0.013

### The differential expression of receptors in HD11 cells upon exposure to endotoxin

**Figure 6:**
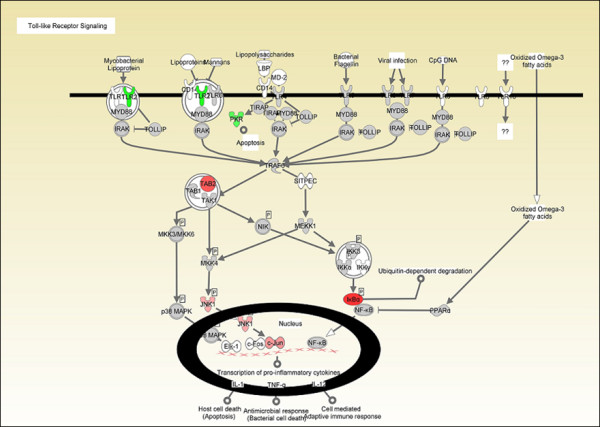
**Toll-like Receptor signalling canonical pathway**. Toll-like Receptor signalling canonical pathway attained by IPA obtained from 4 hours post-stimulation data. Up- and down-regulated genes in red and green, respectively. Experiments were carried out in triplicates.

Fold changes in the DE genes ranged from 1.68^-1 ^to 5.65 at all time points, but *q *values were highly significant (*q *= 0.05) [additional file 2]. We did not detect a significant increase in mRNA level of any *TLR *during the course of exposure; however, *TLR2 *was significantly down-regulated at 4 hps (fold change, 1.65^-1^; *q *= 0.009756) (Fig. 6). Interestingly, *NLRC5 *(CARD domain containing, NLR family, RCJMB04_18i2), an intracellular receptor, in HD11 cells treated with endotoxin for 4 hours (fold change, 1.4; *q *= 0.047) was induced in the present study. Similar to *TLR*s, the *NLR*s recognize pathogen associated molecular patterns that are expressed by bacteria and then activate translocation of NFκB from the cytosol to the nucleus. *NLRC5 *was responsive to endotoxin; however it was not included in either gene networks or functional groups [additional file 1]. Despite accumulating research data, the exact molecular mechanism of *NLR *activation and the initiation of signaling cascades in mammals are not yet fully defined [21]. The data of the present study, however, clearly identify a role for *NLRC5 *in chicken macrophage response to endotoxin.

## Discussion

We did not detect any significant up-regulation in the mRNA levels of *TLR3, TLR4, TLR5, TLR6, TLR7, TLR15, LOC768669 *(similar to TLR1)/*TLR16/TLR6 *(eukaryotic homology group) in the microarray results of this study. Only *TLR2 *showed a significant change in the mRNA level and was slightly, but significantly, down-regulated in stimulated cells. In contrast, *NLRC5 *(NLR family, CARD domain containing 5), was significantly up-regulated. The downregulation of TLR2 might be considered as a result of NLRC5 activation after endotoxin stimulation. The inhibitory effects of NLRC5 on inflammatory pathways have recently been reported [22].

Chicken Tumor Necrosis Factor (*TNF*) alpha gene has not been identified in the chicken genome yet. Interestingly, our study reports differential expression of three TNFalpha-related genes after 1 hour endotoxin exposure, including *TNFAIP3 *(Tumor Necrosis Factor, alpha-induced protein 3), *TNIP2 *(TNFAIP3 interacting protein 2), and *TRAF3 *(TNF receptor-associated factor 3) genes, thus providing additional evidence of existence of genes with TNFA function in chickens. There are still numerous cytokines to be identified, because of limitations in the completeness of the chicken genome assembly [23].

Inflammatory response to infections and tissue injuries is a complex process. Because the inflammatory response causes tissue damage and significant changes in tissue physiology, it must be tightly regulated. The genes that encode antimicrobial effectors do not cause tissue damage and are important for the macrophage early host defence [24]. The differential expression of antimicrobial effectors, but not other functional categories at 4 hps, may be an indication of a self-tolerance mechanism that was developed by chicken macrophages.

Mammals and birds diverged 300 million years ago. There are evolutionarily conserved regions on the chromosomes of both classes [25] such as Toll-like receptor (TLR) encoding genes [26]. Specific receptor for LPS is TLR4 in mammals. It can make the combined use of MyD88-dependent and -independent signalling pathway, while chicken TLR4 cannot. Key components involved in mammalian MyD88-independent TLR4 signalling are LPS Binding Protein (LBP), the lipid scavenger protein CD14, and the intracellular adaptor molecule TRAM [27]. Examination of the chicken genome demonstrates no orthologs for these proteins, with the exception of a CD14-like molecule.

Based on the similarities among the experimental designs, we compared our findings with those reported by Bliss et al., (2005) and Zhang et al., (2008) using the NCBI GenBank gene expression omnibus (GEO) repository, series accession number [GSE1794] [17,15]. Our comparison included inflammatory response genes which were classified by Ingenuity Pathway Analysis software. *IL1B *and *IL8 *genes were the only genes that showed upregulation in all three studies. Zhang et al., (2008) expression data reported upregulations for *CCL4 *and *CD83 *genes, while our results were in concordance with Bliss et al., (2005) on the expressions of *TRAF6, c-fos*, and *TLR1/16/6 *genes [17,15]. The rest of the compared genes did not show a commonality in the expression, probably due to the differences among the experimental conditions, exposure time and the stimulator.

One of the promoter regulatory elements that mediates LPS response in human monocytes is the TPA (12-O-tetradecanoylphorbol 13-acetate)-response element (TRE). The transcription factors that bind to TRE sites are called the Activator Protein 1 (AP-1) complex. They are composed of both the Jun (c-Jun, JunB, and JunD) and Fos (c-Fos, FosB, Fral, and Fra2) families [28]. AP-1 activity is regulated by induced transcription of *c-Fos *and *c-Jun *and/or by posttranslational modification of their products in mammals. c-Jun is ubiquitously present in cells in an inactive form that can be activated through phosphorylation by c-Jun N-terminal kinase (JNK), which belongs to the MAP kinase family [29]. Kogut et al., (2008) demonstrated that chicken heterophils stimulated with flagellin and LPS exhibited a significant increase in DNA binding by the AP-1 family members c-Jun and JunD [30]. The current study shows significant induction of *MAPK8 *at 4 hps (1.32 fold; *q *< 0.04) that may have activated *JUN *at 4 hps. Exposure of cells to various stimulants results in the release of NFκB from inhibitor IκB that controls NFκB activity. Signals activate NFκB by targeting IκB for proteolysis [31]. IκB is degraded by a phosphorylation-dependent ubiquitination process. Our data report the significant up-regulation of *IRAK2 *gene and the genes that are involved in the ubiquitination process to activate NFκB (Table 5).

Although QPCR results showed higher fold changes than the microarray data, they supported the microarray data as to direction of change for the majority of the genes tested. QPCR is able to measure much larger expression changes than microarray because of the larger dynamic range of QPCR experiments [32]. Moreover, the two methods require and use different normalization methods [33].

## Conclusions

We investigated the transcriptional response after in vitro exposure to endotoxin from *Salmonella typhimurium*-798 of the chicken macrophage cell line HD11 as a model for chicken host response to bacteria. Both QPCR and microarray analysis were performed to define the magnitude and the kinetics of innate immune response. Our data showed a strong macrophage response to endotoxin at 4 h post-stimulation, which decreased dramatically by 8 h post-stimulation. About two-thirds of the significantly differentially expressed genes were up-regulated. The *NFKBIA, IL1B, IL8*, and *CCL4 *genes were consistently induced at all time points after endotoxin treatment, demonstrating their important role in response to *Salmonella*. Additionally, the up-regulation of *JUN *and *MAPK8 *at 4 h post-stimulation shows chicken cells use this additional pathway to induce an immune response through the AP1 transcription factor. Although none of the TLRs were upregulated after endotoxin stimulation, the CARD5 domain containing NOD like Receptor 5 (*NLRC5*), an intracellular receptor, was upregulated in response to *Salmonella *endotoxin. To our knowledge, this is the first report of the *NLRC5 *induction by bacterial membrane components in chickens. The recognition of *Salmonella typhimurium*-798 endotoxin by chicken macrophages clearly caused multiple signalling cascades to be initiated and resulted in many gene expression changes. The number of DE genes decreased by 96% from 4 hours to 8 hours post stimulation. This suggests that chicken macrophages quickly return to homeostasis after response to endotoxin-caused shock. This study enhances knowledge on the chicken macrophage transcriptional response to endotoxin by elucidating the complex gene networks involved in the chicken inflammatory response and reports the novel involvement of *NLRC5*.

## Methods

### Cell Culture and Stimulation

The chicken HD11 macrophage cell line [11] was cultured in RPMI 1640 medium (Sigma) supplemented with 10% heat-inactivated newborn calf serum, 2 mM glutamine, 1 mM sodium pyruvate, 0.1 mM non-essential amino acids, 100 U/ml penicillin, 100 μg/ml streptomycin, 10 mM HEPES and 5 × 10^-5 ^M 2-mercaptoethanol (pH 7.3) at 41°C and 5% CO_2_. Cells were plated in 75 cm^2 ^tissue flasks (Cellstar, Greiner Bio-one) and cultures were split every 3 days. Cell viability was > 90% by trypan-blue exclusion (Sigma-Aldrich Co.). Prior to stimulation with endotoxin dissolved in Phosphate Buffer Saline, cells were cultured at an initial density of 2.8 x10^6 ^cells/flask into 25 cm^2 ^tissue flasks and kept overnight in the incubator, then stimulated with 0.0 (vehicle treated), 0.1, 1.0, 10.0 ug/ml endotoxin which was isolated from Salmonella *typhimurium*-798 utilizing the aqueous butanol-1 extraction procedure as described by Morrison and Leive 1975 [34]. Cells were collected at 1, 2, 4, and 8 hours after endotoxin stimulation.

### RNA Isolation, DNase Treatment and QPCR Experiments

Total RNA was isolated from pooled samples (3 individual 25 cm^2 ^flasks per treatment, 3 treatment replicates per each of three treatments performed on different days) using RNAquous^© ^(Ambion, Austin, TX) according to manufacturer's instructions. The mRNA expression levels of *TLR15, IL1B, IL6, IL10, IL8*, and *IFNG *were determined by quantitative real-time RTPCR, using QuantiTect SYBR Green RT-PCR (Qiagen, Waltham, MA). Each RT-PCR reaction was run in triplicate for each sample and consisted of either 50 ng or 75 ng total RNA, 12.5 ml QuantiTect SYBR Green master mix, 0.25 ml QuantiTect RT mix, forward and reverse primers, and RNAse-free water for a final volume of 25 ml. The QPCR primer sequences have been previously published [35-37].

The QPCR reactions were performed on an Opticon 2 (MJ Research Inc., Waltham, MA). An initial 50°C step for 30 min was followed by 95°C for 15 min and 40 cycles (94°C for 15 s, 59°C for 30 s, and 72°C for 30 s, for denaturation, annealing, and extension, respectively) for all PCR amplifications. Gene slopes were determined with serial dilutions differing by 10-fold. A melting curve from 60 to 90°C with a reading at every 1°C was also performed for each individual RT-PCR plate. Adjusted cycle threshold (C(t)) values were calculated as follows:

40 - [C (t) sample mean + (C(t) 28 s median -C(t) 28 s mean)] * (gene slope/28 s slope) for all genes except *IFNG*. The threshold of 40 cycles was raised to 45 cycles for *IFNG*, because most adjusted cycle numbers were greater than 40. Mean adjusted C(t) values of each triplicate of assays were used in statistical analysis. All RNA samples were DNase treated with DNA-Free (Ambion, Austin, TX) according to manufacturer's instructions before QPCR. The fold changes in mRNA levels were determined as follows:

Δ*C*(T) non-stimulated = *C*(T) target gene non-stimulated-*C*(T) 28 s non-stimulated. Δ*C*(T) stimulated = *C*(T) target gene stimulated-*C*(T) 28 s stimulated. The fold change in mRNA = 2^(ΔC(T) non-stimulated-ΔC (T) stimulated)^

### Statistical Analysis of QPCR Data

The mRNA expression levels for each gene were analyzed with the JMP software (SAS Institute, Cary, NC) ANOVA model. The main fixed effects were time (1, 2, 4, 8 hours) and ST-798 endotoxin dose (0.0, 0.1, 1.0, 10.0 μg/ml) and the interaction of these effects. Multiple comparisons of least squares (LS) means for dose and time effects were determined by Tukey-Kramer honestly significant differences test using JMP statistical software (SAS Institute, 2005). *P *< 0.05 was considered as statistically significant [38].

### Microarray Statistical Analysis

The microarray experiment was conducted using three replications. The first two replications each used one experimental unit and one Affymetrix GeneChip for each of the eight combinations of endotoxin dose (treated vs. control) and time (1, 2, 4, or 8 hours after treatment). The third replication was analyzed with four GeneChips for four endotoxin-treated experimental units measured at 1, 2, 4, and 8 hours after treatment, respectively. Data are deposited in the NCBI GenBank gene expression omnibus (GEO) repository http://www.ncbi.nlm.nih.gov/geo/info/linking.html, series accession number is GSE23881. Data were normalized and expression measures computed using the Robust Multiarray Average (RMA) method [39]. A linear model with fixed effects for replication, endotoxin dose, time, and interaction between dose and time were fit to the expression data for each gene using the R package limma [40,41]. As part of each linear model analysis, *P*-values were obtained for the test for dose-by-time interaction, the test for changes over time within endotoxin dose groups, and the test for a dose effect at each time point. The *P*-values for each test were converted to *q*-values for false discovery rate estimation using the method of Nettleton et al. (2006) [42]. The fold changes from microarray data are presented as log base 2.

### Gene Network Analysis

Probe set gene names were downloaded from http://www.affymetrix.com. Construction and statistical significance of gene networks were performed by using Ingenuity Pathways Analysis (Ingenuity^® ^Systems, http://www.ingenuity.com, henceforth abbreviated as IPA) and by selecting *Gallus gallus *in settings. Statistically significant networks were considered those with a *P *value cut-off of 0.0001. Genes were categorized using IPA. The IPA was also used to identify networks of interacting genes. Genes with q values less than 0.05 were entered into IPA.

## Authors' contributions

CC carried out the experiments and data analysis for QPCR, gene networks (IPA), participated in the design of the study and drafted the manuscript. SJL, CKT, MJW, DN participated in the design of the study. MJW provided endotoxin. DN performed the statistical analysis of microarray. All authors read and approved the final manuscript.

## Supplementary Material

Additional file 1 **Differentially expressed genes clustered by function at each time point**. Click here for file

Additional file 2 **All genes list; fold changes (stimulated vs non-stimulated) and q values by time for DE genes**. Click here for file

Additional file 3 **Functions of molecules (genes) involved in each gene network, by time**. Interaction with all possible molecules including genes with q values higher than 0.05. Click here for file
